# Exploring communication between people living with motor neurone disease and their close persons with healthcare professionals: a longitudinal qualitative United Kingdom study protocol

**DOI:** 10.1136/bmjopen-2026-122001

**Published:** 2026-06-29

**Authors:** Katharine Weetman, Muzeyyen Seckin, Ping Guo, Rumandeep Tiwana, Eleanor Wilson, Cara Bailey

**Affiliations:** 1 Birmingham Medical School, School of Medical Sciences, College of Medicine and Health, University of Birmingham, Birmingham, UK; 2 School of Nursing and Midwifery, Health Sciences, College of Medicine and Health, University of Birmingham, Birmingham, UK; 3 School of Health Sciences, Faculty of Medicine and Health Sciences, University of Nottingham, Nottingham, UK; 4 St Giles Hospice, Whittington, UK

**Keywords:** Patient-Centered Care, Motor neurone disease, Longitudinal studies, PALLIATIVE CARE, Protocols & guidelines, Patient Preference

## Abstract

**Abstract:**

**Introduction:**

Good communication is imperative to high-quality patient care, particularly for achieving positive outcomes in healthcare consultations. Evidence about communication in clinical consultations for people with motor neurone disease (MND) is limited, but the knowledge gap prevents possible improvements to communication skills that are unique to the challenges that people living with MND encounter. This research aims to better understand and improve communication experiences for patients with MND.

**Methods and analysis:**

A 24-month qualitative longitudinal study whereby data collection will explore 10–15 cases of persons living with MND. For each case, we will recruit one patient, one close person/carer and one healthcare professional for separate interviews alongside an observed clinical consultation. The patient will be interviewed three times at approximately 4–6-month intervals over 12 months to explore the changing experiences, communication challenges and impact on quality of life. Close persons and healthcare professionals will be interviewed once. Data will be analysed to draw out patterns within and between cases, incorporating thematic analysis, corpus linguistics and conversation analysis techniques.

**Ethics and dissemination:**

Ethical approval was granted by the Health Research Authority (ref: 26/WM/0026).

A co-design workshop will develop a framework for an educational toolkit for healthcare professionals to improve communication skills with MND patients. Findings will be disseminated to the academic community, healthcare staff and the public to increase reach and impact (academic journals, seminars, conferences, newsletters, community groups and engagement events). We will make recommendations for improvements to policy and practice.

**Trial registration number:**

ISRCTN15571034.

**STRENGTHS AND LIMITATIONS OF THIS STUDY**

- The interdisciplinary multimethod analytical approach and triangulation of multiple participant groups are a strength of this study, which draws on methods from health sciences and applied linguistics.
- The ethical principles of this protocol and study design have been guided by the lived experience of motor neurone disease through patient and public involvement.
- The proposed sample size is relatively small, and there is an anticipated high dropout/loss to follow-up given the 12-month longitudinal design and the disease mortality rate.

## Introduction

### Background

 Motor neurone disease (MND) is a progressive neurodegenerative disease with an average life expectancy from diagnosis of around 2–3 years, but this can vary.^1^,^3^ The incidence of MND in the UK was estimated in a 2022 study by Burchardt *et al*^4^ as 5.69/100 000. There is currently no cure for MND,^5^ and diagnosis is determined by clinical findings alongside the exclusion of mimicking conditions.^2^ Many people living with MND (plwMND) will develop difficulties with verbal communication as the disease progresses due to muscle weakness affecting breathing and speech;^6^,^10^ this can impact how readily and easily a patient may be able to make their wishes and needs known,^7 8^ such as during advance care planning discussions.^11^ Communication has been shown to be a priority for MND patients and their carers,^8^,^1012^ and is related to quality of life.^10 13^ Subsequently, there is an evolving research base^9^ relating to the use, development and evaluation of augmentative and alternative communication for MND patients,^14^ such as eye-gaze technology.^15^ However, there seems to be a reduced focus on low-tech communication methods of support (eg, gesture, boards and pictures).^6^

Good communication between healthcare providers and patients is essential for high-quality care provision and can impact clinical outcomes.^16^,^18^ However, plwMND may experience uncertainty in terms of communication^19^ exacerbated by difficulties in physical mobility and speech. In addition to the physical needs, approximately 20%–24% of plwMND can experience cognitive changes.^20^ Where communications from healthcare professionals are poor, this may add to MND patient and carer emotional distress^21^ and compromise co-ordination^22^ and delivery of care.^8^ There is also evidence that a lack of understanding of treatment options/information communicated for people with MND can limit decision-making and involvement in advance care planning.^23^ Moreover, patients with MND may equate good communication with good healthcare provision.^8^

Studies on communicating an MND diagnosis have generally indicated that this needs to be improved.^24 25^ For MND, end-of-life outcomes are often poor in comparison to other life-limiting conditions.^25 26^ As the condition deteriorates, communication is more difficult, and the person’s cognition and behaviour are impacted^19^ so that people are often unable to participate in making their own decisions at the end-of-life.^10^ plwMND have reported fearing death due to concerns over respiratory distress, choking, communication loss, physical helplessness, dependency on others, loss of dignity and being a burden, which in turn has created suicidal intent.^24^ There are also challenges for close persons (relatives, friends…) impacting on their well-being including increased psychological distress, financial burden and limited education and training to help them as carers.^27^ Health and social care professionals can also experience numerous challenges in providing good quality end-of-life care, which may be a result of a lack of integration of care services or limited knowledge about individual needs due to communication barriers - all resulting in further reluctance of healthcare professionals to initiate discussion about end-of-life.^28^ However, despite the significance of this topic,^8 14^ research dedicated to communication between healthcare professionals and plwMND and their carers is somewhat limited, although the importance of timely, effective, empathic and sensitive communication is signposted in MND research with other foci such as well-being,^21^ decision-making,^10 22^ palliative care^29^ and advance care planning.^7^

MND research^1^ has identified a need for wider dissemination of MND information to healthcare professionals, particularly in relation to end-of-life care.^29^ A lack of knowledge among clinical staff may lead to a lack of or inappropriate adjustment of communication style and consultation to suit patients.^8 30^ A recent study by Paynter *et al*^8^ goes some way to address the gap but highlights the need to explore the impact of communication challenges for plwMND. This research will contribute towards the evidence base of the communication experiences of plwMND. Research is needed to tailor education for healthcare staff on MND-related communication strategies,^8^ which this study will address directly. Despite the direct potential patient benefit,^8 14^ the imbalance of communication-focused MND research is notable when compared with other management considerations.^2 5 24^

## Methods and analysis

### Aim, questions and objectives

Aim: to explore clinical communication experiences of plwMND and their close persons and identify recommendations for healthcare providers so that the communication needs and preferences of these patients are central to clinical care, particularly as the condition deteriorates at the end of life.

Research question: what are the clinical communication experiences of plwMND (and their close persons) with healthcare providers, and do they change through disease progression?

Objectives:

1. Explore the experiences of communications that plwMND and their close persons have with healthcare professionals through interviews over a period (12 months) and progression of illness trajectory, including the end of life.
2. Observe and analyse live interactions between plwMND, their close persons and healthcare professionals to explore the patient–clinician interaction through detailed conversational analysis techniques.
3. Synthesise findings and co-produce recommendations to inform guidance and communication training to ultimately improve communication and patient-centred care that supports care planning at the end of life.

The project launched in September 2025 with recruitment and data collection planned to commence in June 2026. The project will be completed in August 2027.

### Study design

This is a longitudinal study comprising empirical data collection (qualitative) and co-production of a communication toolkit to aid consultations for people with MND. We completed the Consolidated criteria for Reporting Qualitative research (COREQ) checklist by Tong *et al*.^31^ Longitudinal study designs are an established approach in qualitative research^8 32^ as they can provide insights into the patients’ journey from diagnosis to death, highlighting the changing communication needs at different points in the disease trajectory. Data collection will explore 10–15 cases. Each case will aim to recruit one patient, one close person/carer and one healthcare professional for interviews and observations. The patient will be interviewed at three approximate 4–6-month intervals over ~12 months to explore the experiences, communication challenges and impact on quality of life. Close persons and healthcare professionals will be interviewed once.

Each case dataset for the longitudinal study will comprise up to six components:

- Three interviews with plwMND (eg, month 1, 4–6 and 8–12).
- Interview with a carer or someone who supports plwMND (at any time point).
- Observation of clinical consultation.
- Interview with healthcare professional post-observation.

### Theoretical framework

A Critical Realist approach^33^ will be taken to ensure coherence of design, data analysis and impact, as well as informing the understanding of dying as a process affected by interactions between individuals and contexts.^34^ The critical realist ontology acknowledges the complexity inherent in social phenomena. Critical realism enables researchers to use in-depth information collected from qualitative interviewing methods, which we propose here to explain phenomena in the social world.^35 36^ The framework suggested by Brönnimann^37^ will inform study design to ensure the richness and complexity are captured and can be understood. Fryer’s^38^ critical realist approach to thematic analysis will be used to produce nuanced causal explanations of events and enhance reliability and reflexivity in the approach.

### Setting

There are 24 MND Care Centres and networks across England, Wales and Northern Ireland, developed by the MND Association at hospital and hospice sites that run clinics and outpatient appointments. We will recruit from hospices and hospitals within the Midlands in England UK, that provide specialist MND services, including outpatient, inpatient and day case procedures, treatments, clinics and other care and support provision.

### Sampling and sample size

We will sample five specialist MND services provided by hospitals and hospices located in the Midlands of England. To determine the participant sample size, we used the principles of the ‘information power matrix’^39^ and input from our patient and public involvement (PPI). We will purposively sample^40^ 15 adult participants at different stages in the MND trajectory and up to 15 of their carers (eg, family, friends…) and 10 healthcare professionals. We will aim for maximum variation, so that patients and carers from a range of demographic backgrounds will be invited to the study. Ideally, sampling will be evenly distributed across sites, that is, 2–3 participants of each group per site. However, this is unlikely to be practicable, particularly given size and service variation across sites in the Midlands. Therefore, there is no minimum recruitment number, but a maximum of 10 patients and 10 carers is imposed per site to avoid over-representation or the full sample being collected at a single site. The number of patients and carers to be screened and invited is not specified, as this should be ongoing until recruitment is complete. Participants will be sampled in accordance with the selection criteria in Box 1 below. Our inclusion and exclusion criteria are broad to help ensure that certain groups are not needlessly excluded and that recruitment is diverse, fair and equitable.

Box 1 **Study inclusion and exclusion criteria**

**Inclusion criteria for patients:**

- Adults (over 18 years).
- Able to make an informed decision to take part in the study.
- Able to participate in an interview (written and/or spoken).
- Receiving or having received specialist motor neurone disease (MND) service from a participating hospital or hospice.

Inclusion criteria for carers:*

- Adults (over 18 years).
- Able to make informed consent to take part in the study.
- Able to participate in an interview (written and/or spoken).
- Identified as the primary family member or informal carer providing regular support to an individual diagnosed with MND.

*For the purposes of this study, we define a carer as the primary carer or any person (eg, informal carer, friend, relative or close person of a patient) who provides support for daily living activities (eg, cooking) or personal care (eg, washing) but is not directly employed to do so.

**Inclusion criteria for healthcare professionals:**

- Working in a participating site supporting delivery of MND services.

**Exclusion criteria (all):**

- Unable to consent to take part in this study.
- Medically too unwell to participate or otherwise deemed unsuitable for participation by the clinical team.
- Expressed a wish not to be contacted or involved in research.
- Children and young people under the age of 18 years old.

#### Interventions to be measured and outcomes

Not applicable as no outcome measure for this study.

#### Recruitment and data collection

Participants will be screened, selected, and recruited by participating hospital and hospice site staff and/or via the local Research Delivery Network (RDN). This approach may be in person or by telephone. All potential participants will be provided with an electronic and/or hard copy of the invitation letter, participant information sheet and consent form. For patients, a short audio clip will be prepared to accompany the information sheet to offer a more appropriate way to learn about the study for some participants who find reading time-consuming or difficult. The information sheet includes: an introduction to the purpose of the research, information on who is conducting and funding the study, details on what the study is about and what taking part would involve, the process for withdrawal, study benefits and risks, expenses, confidentiality and how the data will be used, data sharing, ethical review details and contact details for further information about the study and support resources. Information sheets will be supplemented verbally to ensure participants receive the necessary information to make informed, specific and voluntary decisions.^35^ Interested participants can then contact the research team directly to take part. Alternatively, interested participants can provide verbal consent to the recruiting/care team for their details to be passed directly to the research team, who will then make contact. This is something we have found efficient and effective in past studies when recruiting through hospices and palliative care services as it reduces the burden of interested participants needing to remember to contact the research team alongside managing their condition and appointments.

Carers and healthcare professionals will be invited to one interview. Patients will be invited to approximately an hour of face-to-face and/or virtual interview(s) with the research team at three different time points to assess and capture changes across the disease trajectory (4–6-month intervals).

#### Participant interviews (all)

Interviews will be semi-structured with the experienced research team (CB, EW, MS, RT and KW) and will seek to elicit the participants’ views on living or caring for someone with MND, the communication needs and experiences of these patients, and how experience and quality of care, with a focus on communication, can be improved (see online supplemental material for interview guides). Participants will be offered interviews via online video (eg, Microsoft Teams or Zoom), phone, or in-person at the patient or carer’s home, hospice or hospital, or another place of their choice so long as it is private and suitable (eg, University). Some of these locations may be subject to availability (eg, clinical rooms).

Interview timing and format will be flexibly arranged to suit the participants’ needs for example, shorter interviews, options for online, telephone, email interviews or in person, gathering data in shorter segments where communication is limited, summary of key points checks,^23^ while also including traditional considerations that is, going at the participants’ pace, building in time to take breaks and approaching interviews with flexibility and compassion. Given the physical challenges and fatigue often associated with MND, we will enable flexible interview techniques that are responsive to participants’ needs.^23^ Additionally, a variety of communication methods, such as verbal communication, gestures, and alternative and augmentative communication (AAC) devices, can be used to ensure participants can engage as comfortably as possible.^2335 41^,^43^

All data collection will be sound/video recorded on a secure device. All transcripts will be checked for accuracy by the research team. Transcripts will be pseudonymised, and direct identifiers will be removed during the checking process and replaced with generic terms, for example, (DOCTOR) to de-identify data. Such identifiers may include, but are not limited to, names, addresses, specific sites and places. Demographic information on participants will be collected separately to monitor our purposive sampling objectives.

Participants can suggest their own close person and healthcare professional to take part to form a ‘triad case’ for exploration of conversations, understanding, meaning and nuances of communication. The ‘triad’ in the case for this research is the patient living with MND, the close person (family/friend/carer) and the healthcare professional (specialist MND staff working in the hospice or hospital). Gaining the perspectives of all three who are involved in clinical interactions is important because they each may have slightly different perspectives on the experience. We acknowledge the importance of including close persons, as their communication needs may differ. We therefore aim to interview participants separately unless they prefer a joint interview.

#### Clinic observations

Non-participant video-recorded observations of communication interactions (eg, clinic appointments, ward round, telephone consultation) will be conducted for ten of the cases to enable a conversational analysis of the consultation. The observation will last for the duration of the clinical encounter (expected no more than 60 mins). The research team will take field notes during clinical observations to support the video recording.

### Consent

The consent process will be adapted to accommodate communication needs.^23 42^ Informed consent for this study will be received verbally and recorded in writing for all participants (hard copy or electronic, based on their preference). This study only involves adult participants with the capacity to provide informed consent; the assessment of capacity will be undertaken by the participating site’s clinical care team. Nonetheless, proxy signing has been designed into this study to allow participation by persons who are able to read and process information and provide informed consent, but may not have the physical ability to complete the written consent form (eg, difficulty in coordination, mobility or writing). The research team acknowledge in some circumstances that electronic completion of the consent form may be difficult or prohibited for those persons with disabilities or health conditions that affect their mobility or small motor skills. Therefore, a participant can nominate a proxy such as a carer or companion (eg, friend, partner, neighbour and family member), healthcare professional, or a member of the research team to tick the boxes and type/sign their name in the signature line for them. The use of proxy signing will be participant-led to ensure autonomy and dignity are prioritised. The consent form has been designed so that proxy signing will be clearly indicated and recorded in all cases that apply. Additionally, ongoing consent, revisited at various stages during the research process,^35^ is important, particularly when participants may experience fatigue during interviews.^23^

There is no minimum time between the invitation to take part in the study, consent, and the data collection. This is because of the need for timing in such life-limiting illnesses to be participant-led so as to reduce the risk of survivorship bias and exclusion of potentially vulnerable participants.^44^ Therefore, in compliance with Health Research Authority (HRA) guidance on a proportionate approach to timeframes,^45^ particularly for this low-risk research, we accept a decision taken at the time of approach. We will make interview slots available as soon as possible after participants have expressed interest in taking part. Furthermore, participant interviews will be arranged at their preference. A maximum upper limit on this period has not been stipulated to flexibly suit participant needs and re-scheduling/cancellation where needed. However, interviews will only be conducted while sites are open for data collection, and no interviews or observations will take place after the study data period has formally closed.

The study materials clearly state that participation is voluntary and participants can decline or withdraw without reason or consequences,^23 46^ and choosing not to take part will not affect participants’ medical rights in any way. Participants can choose to withdraw participation and decline any reminders, without giving a reason, by contacting the hospital/hospice that invited them or the research team. Participants approached by the recruiting healthcare staff who do not want to take part do not need to do anything further. However, given the removal of direct identifiers and that interviews will be analysed collectively across the data set, the withdrawal period is set to end when data collection closes, although specific quotes may still be withdrawn up to publication of study outputs and/or the study ends. It will be possible to comply with this partial withdrawal request after the above withdrawal period, but it will not be possible for participants to withdraw their data in any form after publication/dissemination of study outputs or the study ends (planned 31 August 2027), whichever is first.

### Data analysis plan

Analytical methods will be triangulated and involve techniques from the fields of Health Sciences and Applied Linguistics to include: thematic analysis,^47^ corpus linguistics^48^ and conversation analysis.^49^ Data will be analysed thematically and organised under the derived themes in a framework.^50^ Data will be analysed by the core team (CB, KW, PG, MS and EW) alongside members of the PPI group and further discussed and confirmed at the wider advisory group. Initial coding will be conducted by MS with the support of KW to second code and then reviewed and confirmed by CB. They will openly discuss the context and reasoning behind their coding. Any outstanding disagreements will be resolved by discussion with PG and EW. The shared dialogue with the PPI and advisory group will help align understandings of the critical realism theoretical framework. Combining methods from health sciences and applied linguistics strengthens the approach, as the integration of applied linguistics methods is arguably of direct relevance and importance to the proposed foci of communication.

### Patient and public involvement (PPI)

The proposed research was agreed as an important area of research by the BRHUmB research hub (including 10 public members), established from NIHR Partnership Grants led by CB. In designing the protocol, we worked with an individual with lived experience of MND from the perspective of a carer (friend). We have since recruited two further PPIs with lived patient experience of MND to form a PPI group for the study duration. The PPI group will have eight meetings during the 24-month study (6 PPI meetings and two annual advisory meetings). All meetings will be hybrid to maximise inclusivity. PPI will be reimbursed for their time and any expenses incurred. PPI will advise on study conduct and interpretation of findings throughout the research. Additionally, PPI will be encouraged to engage in mock interviews, data analysis, writing and dissemination. We have allocated a training budget to support equipping our PPI to undertake these activities.

## Ethics and dissemination

### Ethics

Participants will provide informed verbal consent to participate, and this will be recorded in writing through a consent form. Ethical approval was granted by the HRA and the NHS REC in the UK on 31 March 2026, ref: 26/WM/0026. The University of Birmingham is acting as sponsor and data controller for this study. We will also obtain local research approval for each site’s research governance committee. Data protection and confidentiality procedures will be observed.

### Ethical and safety considerations

The full transcripts will be pseudonymised and not be available publicly. Only quotes and data excerpts will feature in outputs to protect the identity of participants and minimise any risk of data being identifiable either directly or through a combination of indirect identifiers. The study datasets will be stored and accessed on the university’s secure devices and servers. Data will be permanently destroyed after 10 years. However, the data and results may be used for secondary analysis for future research and/or research involving modified or different research questions; this may be undertaken by the research team and other researchers. In the latter case, data will only be shared in secure and pseudonymised form. The consent form includes permission for secondary analysis of pseudonymised data.

This research will benefit from the involvement of the expert advisory group and the PPI to develop inclusive and ethical recruitment and participation strategies.^23 46 51^ Members include collaborators from relevant charity organisations alongside healthcare professionals, experts and individuals with lived experience, providing valuable insights for tailoring the study to meet participants’ needs.^23 43^

Taking part in this research should not cause participants any direct harm. However, this research project may be deemed a sensitive or upsetting topic, and participants may leave interviews feeling sad or uncertain. If a participant becomes upset during an interview, the researcher will remind the participant that they can pause or stop the interview at any time. The participant information sheet includes a list of supportive resources, and they will also be signposted to how to access wellbeing support and/or discuss their concerns with their doctor. It is a common occurrence that when interviewing people with serious illnesses or terminal conditions, they will often prefer to have a close person with them during the interview for support;^52^ for example, study participants will therefore have the option to attend the interview alone or with a carer or close person for support. The carers/support person can listen only and/or may be able to take part in the interview (with consent).

Participants will not be directly paid. Each patient and carer will be provided with a £25 high street shopping voucher as a thank you for their time to take part in an interview. Participants can choose to accept the voucher, decline, donate it or give it to somebody else. An anonymised acknowledgement of the contribution of study participants will be provided in all study outputs, such as publications, as a collective statement. Travel and expenses have also been costed as necessary; allowing the option of interviews to take place virtually also reduces the financial burden for participants (eg, removes need to travel). Clinical sites will be reimbursed for staff time and costs in line with RDN guidance.

Longitudinal studies always carry some element of risk to recruitment, particularly with progressive disease. Therefore, there is an anticipated high dropout/loss to follow-up given the 12-month longitudinal design and the disease mortality rate. However, our triad recruitment plan does allow for attrition of around a third of cases, which may be expected to occur due to rapid disease progression, withdrawals or change of interest, complex symptoms (eg, low energy) or death. For this reason, we propose to initially recruit 15 patients with a target of 10 completed triads. Data relating to incomplete triads will still be included in the final analysis (with the limitations acknowledged) to ensure these perspectives are not lost.

This study has sought to ensure and promote equality and diversity from design through to implementation, delivery and dissemination. A purposive sampling strategy^40^ will be used to ensure maximum variation in the sample, so that plwMND from a range of demographic backgrounds, with different life-limiting conditions, and who are at different stages of the end-of-life trajectory will be invited to participate. Participating sites will be deliberately sampled to vary by locality and the associated sociodemographic characteristics of the public population for which the site cares for and seeks to serve for example, indices of deprivation, urban or rural setting, patient age group. We have referred to the guidance and increasing participation toolkit from the Centre for Ethnic Health Research^53^ to ensure widening participation and considerations of equality, diversity and inclusion. Interpreter and support costs have been included to ensure we can recruit and enable opportunities for people to participate if they are non or limited English speakers, use non-verbal communication tools, have learning disabilities or visual or hearing conditions/disabilities to ensure inclusivity and equality in our research strategies. Patient interviews can be verbal (speaking), typing (live Teams chat, Zoom or email), or using other AAC tools, for example, type-to-voice technology, writing boards, etc. This is to acknowledge the varying needs of the patient participants, who may struggle with talking or motor skills. Therefore, interviews can be spoken and/or written.

Our PPI advised on the study terminology and will continue to work with us on the participant-facing documentation to ensure we facilitate inclusion. Participants can contact the research team by email, telephone or the study website for further information.

### Dissemination plan

A co-design workshop at the end of the study will use the findings to inform policy guidance and develop a framework for an educational toolkit for healthcare professionals to improve communication skills with plwMND. The key outputs of the research will be: (1) The co-produced list of recommendations to help inform NICE guidance^54^ to present to policy teams to initiate change and (2) A framework to inform the design of tailored MND-related communication skills toolkit to be implemented into existing training resources for healthcare professionals and members of the public (carers, close persons, volunteers and employers). Accompanying the outputs will be academic peer-reviewed (open access) publications planned as follows: the communication challenges for plwMND and how experiences can be improved (thematic analysis); conversational analysis findings from MND clinic observations; a framework for an educational toolkit for healthcare professionals. We will communicate the findings to participants and participating sites, the study advisory group, healthcare organisations, policy-makers and the public via results summaries, social media, newsletters, University websites and conferences.

## Supplementary material

10.1136/bmjopen-2026-122001 online supplemental file 1

10.1136/bmjopen-2026-122001 online supplemental file 2

## Data Availability

No data are available.
