# Supplementary material for: Exploring communication between people living with motor neurone disease and their close persons with healthcare professionals: a longitudinal qualitative United Kingdom study protocol
Source: BMJ Open. 2026 Jun 29;16(6):e122001. doi: 10.1136/bmjopen-2026-122001 (PMC13331081; doi:10.1136/bmjopen-2026-122001)
Supplement: online supplemental file 1 [file bmjopen-16-6-s001.pdf]

School of Health Sciences, University of Birmingham
RESEARCH TEAM CONTACT NAME AND EMAIL OR PHONE NUMBER

Healthcare Professionals (Interview) GUIDE

I: Interviewer (member of the research team) *Action points Q= Question

I: Hello my name is X. I am a researcher from University of Birmingham and a member of a team that are looking to hear about the experiences of people living with MND (motorneurone disease). We are interested in improving communication and care planning for people and their families and supporting better education for healthcare staff. We can take as long as you need, stop for breaks as you wish, no pressure. Please do let me know if you wish to take a break or rest. I'm very grateful for your time.

Please take this time to re-read and familiarise yourself with the materials you received in advance to include the invitation letter, consent form and participant information sheet.

Do you have any questions? **run through forms details and answer queries as required**

Please could you summarise for me what taking part in this research involves?

*if participant(s) understands project involvement *continue*, if not run through information together and repeat question*

If you are happy to proceed with the interview, we need to complete the consent form and we will begin. This is your choice and you may leave without taking part now or at any time.

form signing/checking/completion

Note to researcher to separately record consent verbally for applicable proxy signature cases.

This interview will be semi-formal. This means that while I do have some planned questions to ask you, *I want to hear about your experience*.

Confirm verbally: "the interview will be recorded and active participation indicates consent to this recording and for your data to be used as outlined in the participant information leaflet. *You may choose not to participate or to end the interview if you do not consent to being recorded or use of your data. The recording will now begin"* ****START RECORDING****

1. Can you tell me about your experiences of communicating and caring for patients with MND?

2. Please describe, in your own words, the content and outcomes of the consultation with patient xxx...

3. How clear do you think the consultation and information was to the patient...

4. Broadly, would you describe this consultation as a better or worse example/experience of your management and communication with a patient with MND and why?

5. Could you comment on the success of your communication in the consultation and impact of this on the patient and carer (if relevant)...

6. In terms of the communication in the consultation, is there any part that you felt could have been improved or did not go well?

School of Health Sciences, University of Birmingham
RESEARCH TEAM CONTACT NAME AND EMAIL OR PHONE NUMBER

- How would you address this?

- Has that happened before?

7. What training did you and do you receive on communicating with patients with deteriorating verbal communication and/or nonverbal patients? How do you feel such training provision can be improved? What would have been useful?

8. Do you think there are any specific and important considerations for communicating with patients with MND?

- Do you have any examples?

9. Do you have experience of using alternative and non-augmentative communication methods within consultations?

- Can you recall times it has worked well or been challenging?

10. Please use this final question as an opportunity to provide any reasons for your answers or share any additional comments. Is there anything further you would like to add relating to communicating with persons with Motor Neurone Disease and their carers?

**Discussion may continue in a relaxed conversational manner and researcher may ask additional questions related to anything else relevant mentioned by the patient*.*

Post interview

- Inform participants that the interview is now finished & stop recording
- Ask them how they found taking part & invite them to ask further questions
- Briefly remind participants what the interview will contribute to
- Thank them for their time

Establish if experiencing any distress as a result of the interview – extend debrief for as long as necessary to re-establish composure
