# Supplementary material for: Exploring communication between people living with motor neurone disease and their close persons with healthcare professionals: a longitudinal qualitative United Kingdom study protocol
Source: BMJ Open. 2026 Jun 29;16(6):e122001. doi: 10.1136/bmjopen-2026-122001 (PMC13331081; doi:10.1136/bmjopen-2026-122001)
Supplement: online supplemental file 2 [file bmjopen-16-6-s002.pdf]

School of Health Sciences, University of Birmingham
RESEARCH TEAM CONTACT NAME AND EMAIL OR PHONE NUMBER

PATIENT (Interview 1) and/or CARER INTERVIEW GUIDE

I: Interviewer (member of the research team) *Action points Q= Question

I: Hello my name is X. I am a researcher from University of Birmingham and a member of a team that are looking to hear about the experiences of people living with MND (motorneurone disease). We are interested in improving communication and care planning for people and their families and supporting better education for healthcare staff. We can take as long as you need, stop for breaks as you wish, no pressure. Please do let me know if you wish to take a break or rest. I'm very grateful for your time.

Please take this time to re-read and familiarise yourself with the materials you received in advance to include the invitation letter, consent form and participant information sheet.

Do you have any questions? **run through forms details and answer queries as required**

Please could you summarise for me what taking part in this research involves?

*if participant(s) understands project involvement *continue*, if not run through information together and repeat question*

If you are happy to proceed with the interview, we need to complete the consent form and we will begin. This is your choice and you may leave without taking part now or at any time.

For joint interviews only: If one or the other of you decide not to take part, the other may continue with the interview, if you wish.

form signing/checking/completion

Note to researcher to separately record consent verbally for applicable proxy signature cases.

This interview will be semi-formal. This means that while I do have some planned questions to ask you, *I want to hear about your experience*.

Confirm verbally: "the interview will be recorded and active participation indicates consent to this recording and for your data to be used as outlined in the participant information leaflet. *You may choose not to participate or to end the interview if you do not consent to being recorded or use of your data. The recording will now begin"* ****START RECORDING****

Let's start with finding out a little bit more about you:

Opening

Q: Please can you tell me briefly about yourself and when you were diagnosed with MND?

Q: Can you share your experience of how your MND diagnosis was communicated to you / your family?

- How did you react, and did it affect how you were treated afterwards?

Potential follow-ups and prompts (not all will necessarily be used or relevant for every interview)

School of Health Sciences, University of Birmingham
RESEARCH TEAM CONTACT NAME AND EMAIL OR PHONE NUMBER

Experience of care services

Q: Can you tell me about how you or the person you are caring for came to be known to the hospice / hospital [specialist palliative care service] and what they have been supporting you/the person you are caring for with?

- Prompts – how often do you see them?

Q. Who else supports you with your care? (e.g community, neighbourhood, mnd centres, outpatient clinics, GP etc)

Q: Thinking back to your recent clinical interactions or discussions, do you feel your needs have changed since your diagnosis? What are the differences / challenges / difficulties?

Communication

Q:Can you tell me in your own words how important communication is to you

Q: There are concerns that communication between plwMND and healthcare professionals is not always good. What are your thoughts on that?

- Do you have an example where communication has been good? Why? What helped?

- Do you have an example where communication was poor or made you upset / or you were misunderstood? Why? What could have been done better in that situation?

Q: In your experience are staff adequately prepared / understand the specific needs of plwMND around communication?

Q: Has anyone ever raised care planning with you for when your condition may deteriorate? (End of life care planning? Advanced care planning?) Is this important to you?

Preferred place and providers of (palliative) care

Q. What do you think about the health and social care services that are supporting you currently? [Prompts include: GP, District Nurse, Specialist Palliative Community Nursing Service, Community Palliative Care Doctor, Hospice-At-Home, Social Workers, Night Services, Support Workers].

Q. What are your current preferences for how you or the person you are caring for are supported currently? Is there any support you would like, but are not getting?

Closing

Q: How do you think the process of communication in clinical interactions (clinics, appointments etc) can be improved?

Q: Is there anything else you would like to talk to me about today related to this study?

**Discussion may continue in a relaxed conversational manner and researcher may ask additional questions related to anything else relevant mentioned by the participants*.*

Post interview

- Inform participants that the interview is now finished & stop recording
- Ask them how they found taking part & invite them to ask further questions

School of Health Sciences, University of Birmingham
RESEARCH TEAM CONTACT NAME AND EMAIL OR PHONE NUMBER

- Briefly remind participants what the interview will contribute to
- Thank them for their time

Establish if experiencing any distress as a result of the interview – extend debrief for as long as necessary to re-establish composure
