# Supplementary material for: Exploring communication between people living with motor neurone disease and their close persons with healthcare professionals: a longitudinal qualitative United Kingdom study protocol
Source: BMJ Open. 2026 Jun 29;16(6):e122001. doi: 10.1136/bmjopen-2026-122001 (PMC13331081; doi:10.1136/bmjopen-2026-122001)
Supplement: Supplementary file 3 [file bmjopen-2026-122001.reviewer_comments.pdf]

PEER REVIEW HISTORY

BMJ Open publishes all reviews undertaken for accepted manuscripts. Reviewers are asked to complete a checklist review form and are provided with free text boxes to elaborate on their assessment. These free text comments are reproduced below.

ARTICLE DETAILS

Title (Provisional)

Exploring communication between people living with Motor Neurone Disease and their close persons with healthcare professionals: a longitudinal qualitative United Kingdom study protocol

Authors

Weetman, Katharine; Seckin, Muzeyyen; Guo, Ping; Tiwana, Rumandeep; Wilson, Eleanor; Bailey, Cara

---

This article was not externally reviewed at BMJ Open. Protocols that have been independently assessed prior to submission to BMJ Open are usually fast-tracked to publication on the grounds that further substantial changes will not be possible. This independent assessment will usually be external, independent review for both a substantial grant award from a non-commercial or government funder and ethics approval.
